# Experimentally Induced Mammalian Models of Glaucoma

**DOI:** 10.1155/2015/281214

**Published:** 2015-05-03

**Authors:** Makoto Ishikawa, Takeshi Yoshitomi, Charles F. Zorumski, Yukitoshi Izumi

**Affiliations:** ^1^Department of Ophthalmology, Akita Graduate University School of Medicine, 1-1-1 Hondo, Akita 010-8543, Japan; ^2^Department of Psychiatry, Washington University School of Medicine, St. Louis, MO 63110, USA; ^3^The Taylor Family Institute for Innovative Psychiatric Research, Washington University School of Medicine, St. Louis, MO, USA

## Abstract

A wide variety of animal models have been used to study glaucoma. Although these models provide valuable information about the disease, there is still no ideal model for studying glaucoma due to its complex pathogenesis. Animal models for glaucoma are pivotal for clarifying glaucoma etiology and for developing novel therapeutic strategies to halt disease progression. In this review paper, we summarize some of the major findings obtained in various glaucoma models and examine the strengths and limitations of these models.

## 1. Introduction

Glaucoma is characterized by progressive and accelerated loss of retinal ganglion cells (RGCs) and their axons [1]. Although the pathogenesis of glaucoma is not fully understood, it is believed that increased intraocular pressure (IOP) is a major contributor even in normal tension glaucoma [2]. In this context, a wide variety of animal models have been developed to study the effect of elevated IOP on the optic nerve and the RGC degeneration.

In general, animal glaucoma models are classified into two categories: natural-occurring models and induced models. A variety of natural-occurring glaucoma models have been described in different animal species including dog (beagle) [3], albino New Zealand rabbit [4], and DBA/2J mice [5–7]. It has been considered that the disturbance or obstruction of aqueous outflow could be the cause of IOP elevation, which induces a loss of RGCs and excavation of the optic nerve in these models [8]. Because naturally occurring glaucoma models are poor in controlling onset and pathological course of the disease, induced glaucoma models have been developed with the aim to create proper conditions for controlled experiments. The earliest models of induced glaucoma were developed in monkeys [9], and IOP elevation was induced by intraocular *α*-chymotrypsin injections [10]. Thereafter, multiple* in vivo* glaucoma models have been developed using laser photocoagulation of the perilimbal region [11], autologous fixed red blood cell (RBC) injection [12] or microbead injection into the anterior chamber [9], cauterization of episcleral veins [13], or hypertonic saline injection into the episcleral veins [14, 15]. Recently,* in vitro* [16] and* ex vivo* glaucoma models [17] have been developed to improve the accuracy and repeatability of experimental conditions and to examine pathological mechanisms especially in the acute phase of the IOP elevation. Hydrostatic pressure is applied to cells cultured on a rigid substrate or to isolated retinal tissues* in vitro* and* ex vivo*. More recently, transgenic mouse glaucoma models, which were genetically modified by the introduction of a foreign DNA sequence into a mouse egg, have emerged [18].

In this paper, we present a summary of experimentally induced mammalian glaucoma models that have been developed and used for the recent study of the different types of glaucoma and discuss limitations and potential use of each model.

## 2. *In Vivo* Glaucoma Models

### 2.1. Laser Photocoagulation of the Perilimbal Region

Multiple studies have used laser photocoagulation which induced sustained IOP elevation in monkeys [11], mice [19, 20], rats [21], and rabbits [22–24]. These models were primarily developed to study retinal IOP-related posterior segment damage. The IOP elevations in eyes treated with laser photocoagulation are thought to result from increased resistance of outflow pathways such as angle closure, trabecular scarring, and obliteration of Schlemm's canal [11]. Gaasterland and Kupfer (1974) [11] applied repeated, circumferential argon laser photocoagulation to the trabecular meshwork in both eyes of each of the five Rhesus monkeys and induced a sustained IOP elevation in seven out of ten eyes by a marked reduction in outflow. The IOP range was between 24 and 50 mm Hg after the 4th treatment and remained elevated by 30 days. The main outcome analyzed to determine whether this experimental ocular hypertension can induce a glaucoma was indicated by the observed development of cupping of the optic disc and by the selective loss of retinal ganglion cells in histopathologic specimens. This model became the standard for laser-induced glaucoma in monkeys [25–29].

The advantage of the primate model is that the monkeys have eyes with similar anatomical characteristics to humans. Although monkeys are excellent animal models for studying human disease, there are several limitations to use monkeys, including ethical and economic factors [30, 31]. Laser photocoagulation requires expensive ophthalmic equipment and highly specialized techniques. Laser photocoagulation sometimes induces the inflammation of the anterior chamber and irreversible mydriasis [28].

In contrast to primate models, there are several advantages of using rodents (mice, rats, and rabbits) in glaucoma research. Rodents are inexpensive and easy to house and handle, their eyes are easy to obtain, and the sample number for studies can be large [32]. Aihara et al. (2003) [19] applied argon laser photocoagulation to the corneal limbus in Black Swiss mice after flattening the anterior chamber by aspiration of aqueous humor and successfully induced persistent elevation of IOP for at least 6 weeks in mouse eyes. The flattening of the anterior chamber appears to bring the trabecular meshwork into closer proximity to limbal areas targeted with the laser and may be useful in enhancing the effect of photocoagulation to obstruct the anterior chamber angle [19]. Significant increases in mean IOP during 4 to 12 weeks were detected in treated eyes [19]. Average IOPs in laser-treated eyes (IOPtx) versus contralateral control eyes (IOPc) during the first 4 weeks and during the entire 12-week study period were 23.4 ± 5.1 mm Hg versus 16.3 ± 2.3 mm Hg and 20.1 ± 3.5 mm Hg versus 16.2 ± 2.4 mm Hg, respectively [19]. Average IOPtx was significantly higher than the average IOPc during both periods (*P* < 0.001) [19]. A treatment response was considered to be a success if either the mean of IOP measurements collected during the first 4 weeks was increased by 30% or more or the mean of all measurements collected during the 12-week study period was increased by 30% or more [19]. During the first 4 weeks, 14 (64%) of 22 eyes had an IOP increase of more than 30% [19]. During the entire 12-week study, 7 (37%) of 19 eyes maintained an IOP increase of greater than 30% [19]. The success rate of IOP elevation after a single procedure is relatively high compared with other glaucoma models. Histologic analysis at the end of the 12-week study showed that the angle was completely closed by the laser photocoagulation treatment [20]. Disadvantages of this method include ocular inflammation induced by laser treatment, flattening of the anterior chamber, and variability of IOP magnitude and duration [19]. In addition, the IOP elevation was not sustained in the treated eyes, slowly declining to baseline by 8 weeks after treatment [19]. In optic nerve cross sections examined by electron microscopy 300 *μ*m posterior to the globe [20], mean axon density and total number of axons in the laser treated eyes were significantly decreased compared with the control eyes.

Other studies applying argon laser photocoagulation to the episcleral and limbal veins in C57BL/6 mice [33, 34] induced IOP elevations lasting for weeks after treatment, with decline to baseline levels approximately 8 weeks after treatment [34]. After laser treatment, mean IOP was increased in the treated eyes from the control mean of 13 ± 1.8 mm Hg to 20.0 ± 2.8 mm Hg at 4 weeks in C57BL/6 mice [33]. Peak IOP was 32 ± 2.5 mm Hg in the experimental group. RGC loss was 16.9% ± 7.8% at 2 weeks (*P* < 0.05) and 22.4% ± 7.5% at 4 weeks (*P* < 0.05) after laser photocoagulation [33]. TUNEL staining showed that there were marked increases in the number of apoptotic nuclei in the ganglion cell layer in the treated eyes [33]. Laser photocoagulation of limbal and episcleral veins also induces transient ocular hypertension in albino CD-1 mice [35]. In albino CD-1 mice, the IOPs measured in operative eyes (27.6 ± 2.6 mm Hg) were significantly elevated above those measured in control eyes (12.3 ± 1.0 mm Hg) 4 hours after laser treatment and remained elevated at the second postoperative day (operative 27.1 ± 1.8 mm Hg versus control 13.4 ± 0.3 mm Hg) [35]. IOPs measured in laser-treated eyes declined to baseline and were similar to IOPs in control eyes by 1 week (operative 15.4 ± 1.3 mm Hg versus 12.4 ± 0.6 mm Hg). Overall, the elevation of IOP is transient in these laser models, and the level of cell loss is modest.

Similar to mice, rats are easy to maintain in the laboratory, and they can be used in large numbers [21, 36–40]. Laser photocoagulation has been applied to the trabecular meshwork alone [41] or the trabecular meshwork and episcleral veins [42] in rats, and the induced IOP elevation results in subsequent glaucomatous damage, including RGC loss [41, 42]. Levkovitch-Verbin et al. [42] induced experimental glaucoma unilaterally in Wistar rats, using a diode laser with wavelength of 532 nm aimed only at the trabecular meshwork (trabecular group) or at episcleral veins (combination treatment group) through the external limbus. IOP was increased in all eyes to higher than the normal mean IOP of 19.4 ± 2.1 mm Hg after the laser treatment [42]. Peak IOP was 34.0 ± 5.7 mm Hg in the trabecular group and 49.0 ± 6.1 mm Hg in the combination group [42]. Mean IOP after 6 weeks was 22.0 ± 1.8 mm Hg in glaucomatous eyes in the trabecular group compared with 25.5 ± 2.9 mm Hg in the combination group [42]. IOP in the glaucomatous eyes was typically higher than in the control eyes for at least 3 weeks [42]. In the combination group, RGC loss was 16.1% ± 14.4% at 1 week (*P* < 0.01), 59.7% ± 25.7% at 6 weeks (*P* < 0.001), and 70.9% ± 23.6% at 9 weeks (*P* < 0.001) [42]. The trabecular group had mean axonal loss of 19.1% ± 14.0% at 3 weeks (*P* < 0.004) and 24.3% ± 20.2% at 6 weeks (*P* < 0.001) [42]. Laser treatment led to closure of intertrabecular spaces and the major outflow channel [42]. The retina and choroid were normal by ophthalmoscopy at all times after treatment. Light microscopic examination showed only loss of RGCs and their nerve fibers [42]. Although continuous IOP elevation over longer periods is ideal, 3 weeks of elevated IOP induces substantial RGC loss and axonal damage of the optic nerve, making the model attractive for most investigations [42].

There are several limitations to using laser-induced ocular hypertension in rats. First, differences in pigmentation of the trabecular meshwork markedly change the effects of laser photocoagulation to increase IOP. Second, repeated laser treatments induce ocular inflammation and corneal opacity [43].

### 2.2. Red Blood Cell or Microbead Injections into the Anterior Chamber

To circumvent the limitations and disadvantages of laser techniques, microbeads were injected into the anterior chamber to induce ocular hypertension in primates [9], pigs [30], mice [44], rats [45], and rabbits [46]. An alternative to microbead injection uses injection of autologous fixed red blood cells (RBCs) into the anterior chamber [12, 47]. Elevations in IOP observed in autologous RBCs- or microbead-injected eyes are thought to result from inhibition of aqueous outflow.

Experimental primate models of chronic IOP elevation were developed by Quigley and Addicks (1980) using autologous fixed RBCs (ghost cells) [12, 47]. Direct obstruction of the trabecular meshwork by ghost cells as well as swelling of trabecular cells following phagocytosis of cellular debris was observed by electron microscopy. The model has the advantages of producing IOP elevation easily (mean IOP, 24 mm Hg to 73 mm Hg) and without associated intraocular inflammation [12]. However, IOP elevations lasted from 2 to 42 days, and the extensive filling of the anterior chamber with ghost cells resulted in poor visibility of the optic disk [12]. Ghost cells are degenerating red blood cells without hemoglobin content. Subsequently, Weber and Zelenak (2001) [48] reported that multiple injections of sterile latex microspheres (2–4 × 10^5^ sterile beads per injection) into the primate anterior chamber are simple and cost effective for inducing chronic IOP elevation [48]. In the treated eyes with multiple injections of latex microspheres, mean IOP was 17.8 mm Hg to 36.7 mm Hg, and peak IOP was 23 mm Hg to 65 mm Hg [48]. Different levels and durations of elevated IOP can be obtained by altering the frequency and number of microspheres injected [47]. This approach has the advantages of producing IOP elevations while preserving visibility of the optic disc, which is necessary for assessment of glaucoma development [48]. Fluorescent polystyrene microbead injection into the anterior chamber of C57BL/6 mice results in chronic IOP elevation (4.6 ± 0.6 mm Hg above control IOP) lasting for at least 3 weeks following a single injection [49]. Cone et al. (2012) [50] maintained the IOP elevation by a combination of polystyrene bead injection followed by viscoelastic solution injection into the mouse anterior chamber. The disadvantages of the mouse model include the relatively small size of the globe, which makes it hard to manipulate.

IOP elevations induced by microbeads have also been described in rat models [40]. In Wistar rats injected with microbeads, IOP elevation persists for two weeks and results in reduced density of the optic nerves [40, 51]. Wistar rats receiving weekly injections of hyaluronic acid show IOP elevation that persists for at least 10 weeks [51].

Taken together, microbead injection models offer a relatively easy technique without special equipment, and the IOP elevation can be modulated with subsequent injections of microbeads or viscous materials. The principal disadvantage is that microbeads can be difficult to retain in the anterior chamber angle after injection. To address these issues, Samsel et al. (2011) [52] developed a technique for induction of ocular hypertension using paramagnetic microbeads. Magnet is used to direct microbead to the anterior chamber angle. These beads have the advantage that they can be directed to the anterior chamber angle in the rodent eye to optimize occlusion of the trabecular meshwork. In this case, the paramagnetic microbeads could be directed to the iridocorneal angle using a handheld magnet [52].

### 2.3. Episcleral Vein Obstruction

Shareef et al. (1995) [15] developed an episcleral vein cauterization model of glaucoma in rat. This method is less invasive than laser photocoagulation and induces no complications in the anterior chamber [53]. Because of its efficacy and accessibility, the majority of the structural and functional studies in experimental glaucoma have used this method [13]. IOP elevations in this model are thought to involve increased outflow resistance [54].

Mouse glaucoma models induced by episcleral vein cauterization exhibit significantly elevated IOP (28 ± 1.5 mm Hg) for up to 4 weeks and loss of RGCs [54]. Photocoagulation of episcleral and limbal veins induces a doubling of IOP lasting for 4 hours in albino CD1 mice [55, 56]. Complications of episcleral vein cautery in mice include thermal damage to sclera, intraocular inflammation, and ocular surface damage.

To compare the effects of IOP elevation on ganglion cell size and death, Vecino and Sharma (2013) [57] used three experimental glaucoma models in rats: (i) injections of latex microspheres into the anterior chamber, (ii) injections of microspheres and hydroxypropylmethylcellulose into the anterior chamber, and (iii) cauterization of three episcleral veins. IOP elevation induced by episcleral vein coagulation was more stable and constant for at least 24 weeks as compared with the other two experimental glaucoma methods. Similar results were observed when the three methods were compared in rats [58] and pigs [44].

Morrison et al. [14] have suggested, however, that pathophysiology in the episcleral vein cautery model differs from the other two ocular hypertension models, and the pattern of RGC death might be different in this model. While axonal degeneration of RGCs is the predominant finding in the other IOP elevation models, episcleral vein cautery appears to produce general RGC loss, indicating the possibility that factors other than IOP elevation might contribute to RGC death in episcleral vein cautery models.

### 2.4. Episcleral Vein Saline Injection

Kipfer-Kauer et al. (2010) [59] have succeeded in inducing chronic IOP elevation in C57BL/6 mice by injection of 1.5 M hypertonic saline into a limbal vein. The hypertonic saline injection group revealed a mean IOP of 9.99 ± 3.3 mm Hg versus 7.42 ± 2.2 mm Hg in the contralateral control eye. Peak IOP in the hypertonic saline injection group was 15.6 mm Hg versus 11.6 mm Hg in the control group. Episcleral vein saline injection causes an increase in the resistance of aqueous outflow channels. To develop chronically elevated IOP in rats, episcleral veins were injected with hypertonic saline in Brown Norway rats, and IOP elevations (7 to 28 mm Hg above control pressure) were sustained after 4 weeks [14]. The anterior chamber angles showed the formation of the peripheral anterior synechia. Electron micrographs of eyes from this model showed glaucomatous damage of RGC axons [14].

The disadvantage of these latter models is the relative difficulty of the induction technique. Insertion of a microneedle into the rat episcleral vein requires considerable training and experience. An additional disadvantage is that the duration of IOP elevation is relatively short and sequential hypertonic saline injections are needed to produce longer lasting IOP changes [60].

Taken together, a wide variety of* in vivo* animal models have been developed to study the effect of elevated IOP on the optic nerve and RGC degeneration. However, the duration of IOP elevation in these models is transient without sequential treatments. In addition, precise control over IOP elevation is difficult, and the timing of induction and progression of glaucoma are usually unpredictable.  Table 1 is the summarization of characteristics of the experimentally induced* in vivo* mammalian glaucoma models.

## 3. *In Vitro* Glaucoma Models

While* in vivo* animal models are necessary to show that a phenomenon occurs in living organisms, experiments in live animals typically involve undefined and uncontrollable factors [43]. Therefore,* in vitro* experimental systems are useful for producing highly controlled experimental conditions to manipulate specific variables contributing to degenerative changes [43]. Recently,* in vitro* glaucoma models that use cells cultured on a rigid substrate have been described [16, 61–72]. These models have used RGCs, optic nerve head astrocytes, or other types of retinal cells and sometimes have been equipped with pressure loading systems.

**Figure 1:**
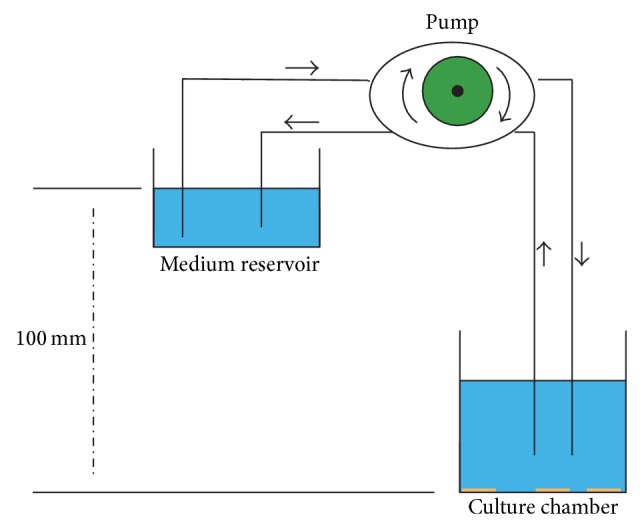
System used for hydrostatic pressure experiments. The culture chamber was filled with medium. The height of the medium reservoir was adjusted to maintain the pressure in the culture. For gas exchange, the medium was circulated by a peristaltic pump, and the pressure was monitored with the pressure gauge. This figure is the modification of Figure  1 of Lei et al. [61] and Figure  1 of Obazawa et al. [73].

**Figure 2:**
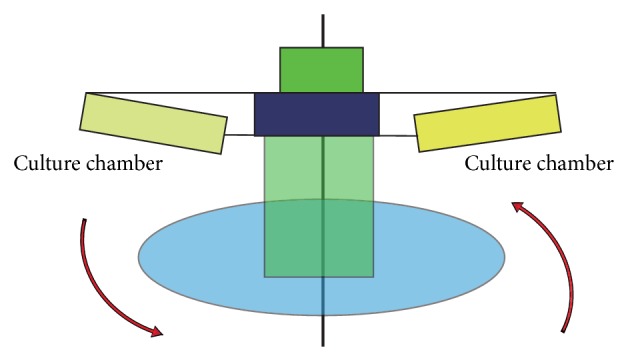
Schema of the centrifugal force loading device. The device is composed of two culture chambers and a motor unit for providing centrifugal force and rotor vessels. The isolated cells are situated at the bottom of the culture dish to become perpendicular to the direction of the centrifugal force and gravity vectors corresponding to a rotation speed. This figure is the modification of Figure 1 of Kashiwagi et al. [74].

The application of hydrostatic pressure induces remarkable changes including enhancement of RGC apoptosis [64, 66], alterations in astrocyte structure [57], cell migration [63], elastin synthesis [68], and production of neural cell adhesion molecules [69]. Obazawa et al. (2004) [73] developed a cell culture system for manipulating hydrostatic pressure to examine the expression of optineurin and myocilin genes in trabecular meshwork cells under normal and hyperbaric conditions (Figure 1). Kashiwagi et al. (2004) [74] examined the survival and morphology of isolated RGCs subjected to centrifugal force loading using the unique device (Figure 2). The device includes a rotating vessel installed within a large incubator (model CPO2-1800; Hirasawa, Tokyo, Japan), a power supply unit, a control unit, and a cooling motor for removing heat generated by the motor installed outside the device. The rotor spins at 1 to 30 rotations per minute (rpm), with a rotation accuracy of 0.01 rpm. The equation for calculating centrifugal force (*F*) is *F* (mm Hg) ≒ 1.12*r* × (rpm/1000) × 750, where *r* is radial distance (in millimeters).

Recently, Yu et al. (2011) [75] developed a more convenient and simple pressure system using T75 culture flasks. An air mixture of 95% air and 5% CO_2_ is pumped into the flasks to obtain the desired pressure.

*In vitro* models are also useful for investigating the role of apoptotic mechanism in RGCs. RGC death induced by IOP elevation involves caspase activation as demonstrated using experimental rat models of glaucoma [76].* In vitro* studies provide strong evidence that apoptosis of retinal neurons induced by different stimuli shares a common caspase cascade [66, 77], which can be inhibited using specific caspase inhibitors [78]. Additionally, Tezel and Yang (2004) [79] applied TNF-*α* or hypoxia to primary cultures of rat RGCs for up to 48 hours and found that inhibition of caspases cannot block RGC death if the mitochondrial membrane potential is lost and cell death mediators (cytochrome c and apoptosis-inducing factor) are released.

Identification of precise cellular mechanisms in glaucoma requires isolation and primary culture of the RGCs. It has been known that* in vitro* experiments using primary cultures of RGCs are difficult to perform, mainly because of the limited yield and the typically postmitotic features of these neurons [80]. Therefore, early postnatal tissues are used in an attempt to optimize cell number and survival in culture. However, there are differences in cell responses between postnatal and adult cells that can limit interpretation of experimental results. In addition, it is difficult to examine interactions between RGCs and other types of cells such as retinal glia in specific mechanisms.

## 4. *Ex Vivo* Glaucoma Models

**Figure 3:**
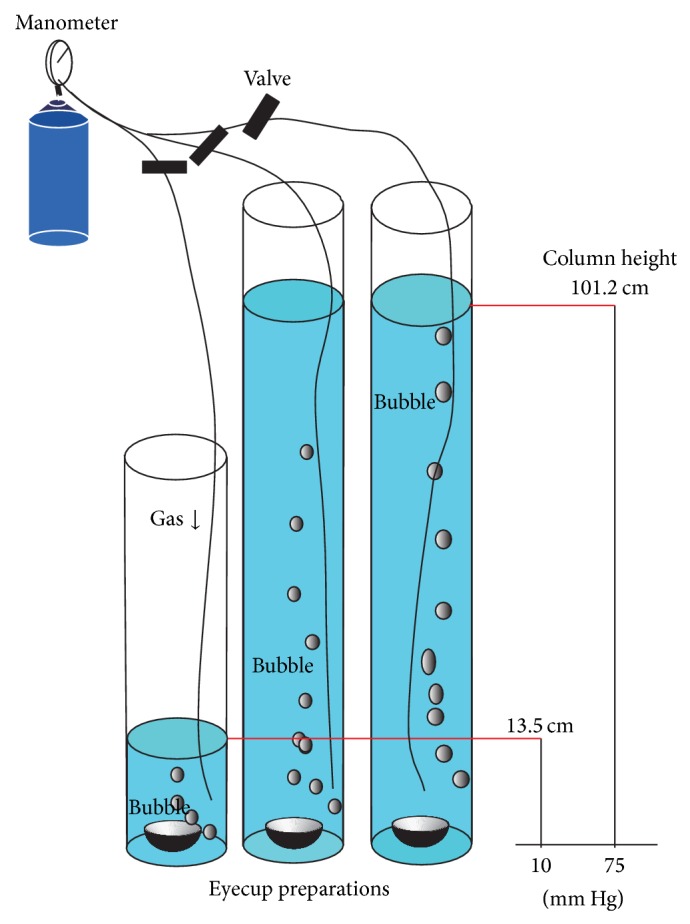
Outline of the experiment using* ex vivo* glaucoma model. Eyecups preparations were sunken to the bottom of a glass cylinder with different heights. Each cylinder was filled with incubation buffer at 30°C for 24 hours. The buffer was bubbled with 95% O_2_-5% CO_2_. Hydrostatic pressure at the bottom of the cylinder was calculated to be 10 mm Hg and 75 mm Hg when a CSF was added to a height of 13.5 cm and 101.2 cm, respectively. This figure is the modification of Figure  1 of Ishikawa et al. [17].

We recently developed a new* ex vivo* experimental model for acute glaucoma that involves incubating rat retinal segments under hydrostatic pressure at the bottom of a deep cylinder [17] (Figure 3). Acute high pressures can induce retinal ischemia clinically and in* in vivo* glaucoma models [81, 82]. The* ex vivo* hydrostatic pressure model excludes the influence of ischemia and can thus allow examination of the direct effects of hydrostatic pressure on the otherwise intact retina, including changes in gene and protein expression [83, 84].

While the* ex vivo* system produces reliable results, we note several limitations. Survival factors for retinal neurons supplied from the blood stream or axonal transport are eliminated in* ex vivo* preparations, and the incubation period is limited by the duration in which the tissue can be kept alive. The advantages of this model include a higher degree of control over experimental variables and better preservation of neuron-neuron and neuron-glial interactions that are possible in dissociated cell preparations.

Of interest in this* ex vivo* model is the finding that axonal swelling in RGCs is induced in a pressure-dependent manner. In the central nervous system, activation of neuronal glutamate receptors induces swelling of cell bodies and dendrites [85–88] and also produces Na^+^-dependent blebs in acutely isolated hippocampal neurons [89]. This swelling is caused by the influx of Na^+^ and Ca^2+^ and the passive redistribution of chloride and water across neuronal membranes [88–90]. Similar events occurring in axons could contribute to the findings observed in the* ex vivo* glaucoma model. Because administration of glutamate receptor antagonists attenuated the axonal swelling, we hypothesize that glutamate-mediated changes contribute to axonal swelling under hyperbaric conditions.

To determine whether increased pressure triggers release of ATP, Reigada et al. (2008) [91] loaded air or nitrogen pressure to* ex vivo* bovine eyecups [91]. One milliliter of buffer solution was added to the bottom of each eyecup and the lid was sealed. Air or nitrogen was injected from a syringe until the pressure reading by digital manometry reached the desired level. When appropriate precautions were taken, pressure levels remained constant throughout the experiment. ATP released from retinal cells diffuses into the vitreous humor. Vitreous humor from each eyecup was collected, and ATP concentrations were determined. Elevated pressure led to an increase in extracellular ATP. This excess extracellular ATP may serve as a link between increased pressure and RGC death in acute glaucoma, suggesting a possible role for ATP in neuronal damage accompanying increased intracranial pressure.

Table 2 is the summarization of characteristics of the experimentally induced* in vitro* and* ex vivo* mammalian glaucoma models.

## 5. Genetically Modified Mouse Glaucoma Models

There are numerous mouse models in which glaucoma-like pathology occurs as a result of genetic mutations [43, 92]. A main advantage of these glaucoma models is higher reproducibility of responses following IOP elevations compared to surgically induced models [42]. Because there is significant conservation in mouse and human genomes, the generation of mice with specific mutations identified in human glaucoma can be useful for understanding pathogenesis [43]. We briefly review studies that have utilized genetically modified mouse models to examine the roles of different genes in the glaucoma pathogenesis.

### 5.1. GLAST and EAAC-1

Normal tension glaucoma (NTG) is a condition in which clinical features are largely identical to those seen in primary open angle glaucoma (POAG) except for the relatively low IOP (<22 mm Hg). The pathophysiology of RGC degeneration and optic nerve damage in NTG remains unclear. To explore possible pathways of RGC degeneration, genetically modified mice with normal IOP have been used as models of NTG. Harada et al. [18] showed that mice with deficient expression of the glutamate transporters, GLAST or EAAC-1, demonstrate spontaneous RGC death and optic nerve degeneration without elevated IOP. In GLAST-deficient mice, administration of a glutamate receptor antagonist prevented RGC loss, indicating that GLAST helps to prevent excitotoxic retinal damage. Additionally, GLAST helps to maintain levels of the antioxidant glutathione in Müller cells by transporting glutamate, the substrate for glutathione synthesis, into the cells. Taken together, it appears that GLAST deficiency leads to RGC degeneration by both excitotoxic and oxidative stress mechanisms.

GLAST deficient mice were also used to investigate ASK1 deficiency on RGC death [93]. Apoptosis signal-regulating kinase 1 (ASK1) is a mitogen-activated protein kinase (MAPK) that plays an important role in stress-induced RGC apoptosis. Loss of ASK1 had no effects on the production of glutathione or malondialdehyde in the retina or on IOP. Tumor-necrosis-factor (TNF) induced activation of p38 MAPK and production of inducible nitric oxide synthase were also suppressed in ASK1-deficient Müller glial cells and RGCs, suggesting that ASK1 activation is involved in NTG.

### 5.2. *CYP1B1*

*CYP1B1* is a gene implicated in congenital glaucoma and codes the enzyme, cytochrome P450, family 1, subfamily b, polypeptide 1 [94, 95]. The role that* CYP1B1* plays in the pathophysiology of glaucoma and the development of anterior chamber anomalies is not known. Nonetheless,* CYP1B1*-deficient mice exhibit abnormalities in their aqueous drainage system that are similar to those reported in human angle-closure glaucoma patients [96]. In contrast, other studies using* CYP1B1*-null mice revealed no evidence of IOP elevation [97]. Although* CYP1B1* knockout mice do not develop elevated IOP, they have abnormalities in their aqueous drainage system, small or absent Schlemm's canal, defects in trabecular meshwork, and peripheral anterior synechia of the iris [97]. A mouse model with mutations in both* CYP1B1* and* Tyr* was also developed and revealed that* Tyr* mutation modifies the phenotype associated with inheritance of mutant orthologs of* CYP1B1* and* Foxc1*, both of which have been shown to be involved in human angle-closure glaucoma [96, 97].

### 5.3. Alpha-1 Subunit of Collagen Type 1

More recently, a transgenic mouse model of POAG has emerged. This mouse model has a targeted mutation in the gene for the alpha-1 subunit of collagen type 1 and demonstrates progressive loss of RGC axons induced by IOP elevation [98]. Organization of the drainage structures seems normal in this model.

### 5.4. Myocilin

The myocilin gene (*Myoc*) encodes a secreted glycoprotein. Tyr437His mutation in* Myoc* leads to severe glaucoma in humans [99], and the mouse Tyr423His mutation corresponds to this human mutation [100]. Tyr423His* Myoc* mice demonstrate progressive degenerative changes in the peripheral RGC layer and optic nerve, with normal organization of aqueous drainage structures [101]. It has been suggested that mice expressing mutated mouse or human* Myoc* in the trabecular meshwork have characteristics of POAG [101, 102]. By contrast, expression of the mutated* Myoc* allele (Tyr423His) specifically in the iridocorneal angle does not lead to IOP elevation and does not produce degenerative changes in the retina [103]. These differences might be explained by differences in the levels of mutated* Myoc* expression as well as by differences in genetic background [103].

## 6. Conclusions

This paper describes animal models used in glaucoma research. These animal models are essential to elucidate the natural course of the disease and to develop novel therapeutic approaches. However, glaucoma is a disorder with complicated pathogenesis that is far from being completely understood. Since the mechanisms of glaucoma differ among animal models, the selection of an animal model should be based on experimental needs and the hypothesis being tested. Experimentally, induced* in vivo* models have the advantage of studying certain changes in glaucoma in a living animal. However, the duration of IOP elevation in these models is transient without sequential treatments. In addition, precise control over IOP is difficult, and the timing of induction and progression of glaucoma are often unpredictable. While* in vivo* animal models are necessary to demonstrate that a phenomenon occurs in living organisms,* in vivo* animal experiments usually include undefined and uncontrollable factors. For this reason,* in vitro* systems have been useful for conducting highly controlled experiments in specific contexts.* In vitro* experiments using primary cultures of RGCs are not easy to perform, however, mainly because of the limited cellular yield in adult animals and the typically postmitotic feature of RGCs. Thus, early postnatal tissues are used in order to optimize cell number and survival in culture. It is important to note, however, that there are major differences in cell responses to external stimulation between postnatal and adult cells. In addition, it is difficult to examine the interaction between RGCs and other types of cells such as retinal glia under these conditions. Recently developed* ex vivo* models for acute glaucoma involve incubating rat retinal segments under hydrostatic pressure at the bottom of a deep cylinder. The* ex vivo* hydrostatic pressure model excludes the effects of ischemia and allows studying of the direct effects of pressure on the retina. Additionally, this model includes higher degrees of control over experimental variables and better preservation of neuron-neuron and neuron-glial interactions that are possible in dissociated cell preparations.* Ex vivo* models are limited by the absence of survival factors supplied by blood or axonal transport, and the incubation period is time limited. Going forward, it is likely that genetic models developed to test specific hypotheses will provide valuable information on pathophysiology and potentially lead to the discovery of new therapeutic targets. By using these animal models, we hope to continue to improve glaucoma prevention and treatment.

## Figures and Tables

**Table 1:** Experimentally induced *in vivo* mammalian glaucoma models.

*In vivo* glaucoma models	Species	Main papers	Main outcomes measured	Cost	Limitation
Laser photocoagulation of the perilimbal region	Monkey	Gaasterland and Kupfer (1974) [11]	IOP, cupping, HR, HI, OF	Expensive (laser equipment)	Ocular inflammation Irreversible mydriasis Technical difficulties
	Mouse	Aihara et al. (2003) [19] Mabuchi et al. (2003) [20]	IOP IOP, HR		Ocular inflammation Variability of IOP Technical difficulties
	Rat	Levkovitch-Verbin et al. (2002) [42]	IOP, HR, HI		Hyphema, corneal opacity Ocular inflammation

RBC injections into the anterior chamber	Monkey	Quigley and Addicks (1980) [12, 47]	IOP, HR, HI, OF		Low visibility of optic discs by accumulation of RBC or microbeads
Microbead injections into the anterior chamber	Rat Mouse	Weber and Zelenak (2001) [48] Pang et al. (2005) [40] Sappington et al. (2010) [49]	IOP, HR, HI IOP IOP, HR, HI	Not expensive	
Hyaluronic acid injection into the anterior chamber	Rat	Moreno et al. (2005) [51]	IOP, HR, HI, ERG		

Episcleral vein obstruction	Rat	Shareef et al. (1995) [15]	IOP	Not expensive	Scleral thermal burns (RGC death patterns may be different from those of other glaucoma models)
	Mouse	Ruiz-Ederra and Verkman (2006) [54]	IOP, HR, OF		

Episcleral vein saline injection	Rat Mouse	Morrison et al. (1997) [14] Kipfer-Kauer et al. (2010) [59]	IOP, HR, HI IOP, HR	Not expensive	Technical difficulties

IOP: IOP measurement, cupping: assessment of optic disc cupping, HR: histological assessment of retinal nerve fibers and optic discs, HI: histological assessment of the iridocorneal angles, OF: outflow facility, ERG: electroretinography.

**Table 2:** Experimentally induced *in vitro* and *ex vivo* mammalian glaucoma models.

	Species	Main papers	Pressure (mm Hg)	Duration (hours)	Main outcomes measured
*In vitro* glaucoma models					
Hydro pressure model	Porcine	Obazawa et al. (2004) [73]	3, 33	12, 24, 48, 72	Optineurin and myocilin expression in trabecular meshwork cells
Centrifugation model	Rat	Kashiwagi et al. (2004) [74]	16, 28, 33	24, 48	Expressional changes in mRNA in RGC and retinal glia

*Ex vivo* glaucoma models					
Hydro pressure model	Rat	Ishikawa et al. (2010) [17]	10, 25, 50, 75	24	Histology of the retinal nerve fiber, Glutamine synthetase activity
Gas pressure model	Bovine	Reigada et al. (2008) [91]	20–100	0.5	Retinal ATP release
